# Cardiorenal Protective Effects of Finerenone in Patients with Type 2 Diabetes Mellitus

**DOI:** 10.26502/aimr.0241

**Published:** 2026-05-02

**Authors:** Talia Radparvar, Ilana Radparvar, Devendra K. Agrawal

**Affiliations:** 1 Taft Charter High School, Woodland Hills, CA 91364 USA; 2 Department of Translational Research, College of Osteopathic Medicine of the Pacific, Western University of Health Sciences, Pomona, California 91766 USA

**Keywords:** Cardiovascular disease, Chronic kidney disease (CKD), CKM syndrome, Endothelial dysfunction, Finerenone, Insulin resistance, Oxidative stress, Renin-angiotensin-aldosterone system, Type 2 diabetes mellitus (T2DM), β-cell dysfunction

## Abstract

Type 2 diabetes mellitus (T2DM) is a chronic metabolic disorder characterized by insulin resistance and progressive β-cell dysfunction, resulting in persistent hyperglycemia and long-term vascular complications. The global prevalence of T2DM continues to rise and is strongly associated with cardiovascular disease, chronic kidney disease, and the broader cardiovascular–kidney–metabolic (CKM) syndrome. This review summarizes the pathophysiology of T2DM, with emphasis on insulin resistance, endothelial dysfunction, oxidative stress, and activation of the renin–angiotensin–aldosterone system, all of which contribute to progressive end-organ damage. Chronic hyperglycemia further promotes inflammatory and fibrotic pathways that accelerate diabetic kidney disease and atherosclerotic cardiovascular disease. Finerenone, a non-steroidal mineralocorticoid receptor antagonist, has emerged as a therapeutic option for patients with T2DM and chronic kidney disease. Clinical trial data demonstrate that finerenone reduces albuminuria and provides cardiovascular and renal protection by reducing inflammation and fibrosis. Its use is associated with a generally acceptable safety profile, although hyperkalemia remains an important adverse effect requiring routine monitoring. Overall, T2DM is a multifactorial disease that requires comprehensive management targeting metabolic dysregulation as well as inflammatory and hormonal pathways. Finerenone represents an important advancement in cardiorenal protection. Understanding the interconnected mechanisms underlying CKM syndrome is essential for improving long-term outcomes in individuals with T2DM.

## Introduction

Type 2 Diabetes Mellitus (T2DM) is an increasingly prevalent chronic disease affecting populations worldwide, driven by a combination of genetic, lifestyle, and metabolic factors. Indeed, there are significant impact of nutrition, gut microbiota, epigenetic factors, apolipoproteins, and adipokine dysregulation in metabolic syndrome, including type 2 diabetes mellitus in the heart, kidney, liver, musculoskeletal system and other organs [1–6]. Underlying cellular and molecular mechanisms may include inflammation, galectin molecules, adipokine dysregulation, vitamin D deficiency, and others leading to the pathogenesis of metabolic syndrome and insulin resistance [7–10].

Current projections indicate that its prevalence will continue to rise, reaching approximately 10.8% of the population in the United States by 2050 [11]. T2DM is the most common form of diabetes and affects hundreds of millions of people globally. In the United States, the number of individuals diagnosed with T2DM is expected to continue increasing, making it a major public health concern due to its association with serious complications such as cardiovascular disease and chronic kidney disease [12].

Over time, T2DM causes widespread damage throughout the body, including chronic inflammation, endothelial dysfunction, and vascular injury, which can ultimately lead to chronic kidney disease (CKD) and cardiovascular disease. This condition is a complex metabolic disorder characterized by persistently elevated blood glucose levels [12]. It develops primarily due to two key defects: insufficient insulin production caused by pancreatic beta-cell dysfunction, and impaired insulin action due to insulin resistance in peripheral tissues.

For normal glucose homeostasis, insulin production and insulin sensitivity must be tightly regulated to meet metabolic demands. This requires precise coordination of insulin synthesis, secretion, and cellular uptake. Disruption in any of these processes leads to metabolic imbalance and contributes to the development and progression of T2DM [13].

### An Overview of Type 2 Diabetes Mellitus

**Figure 1:**
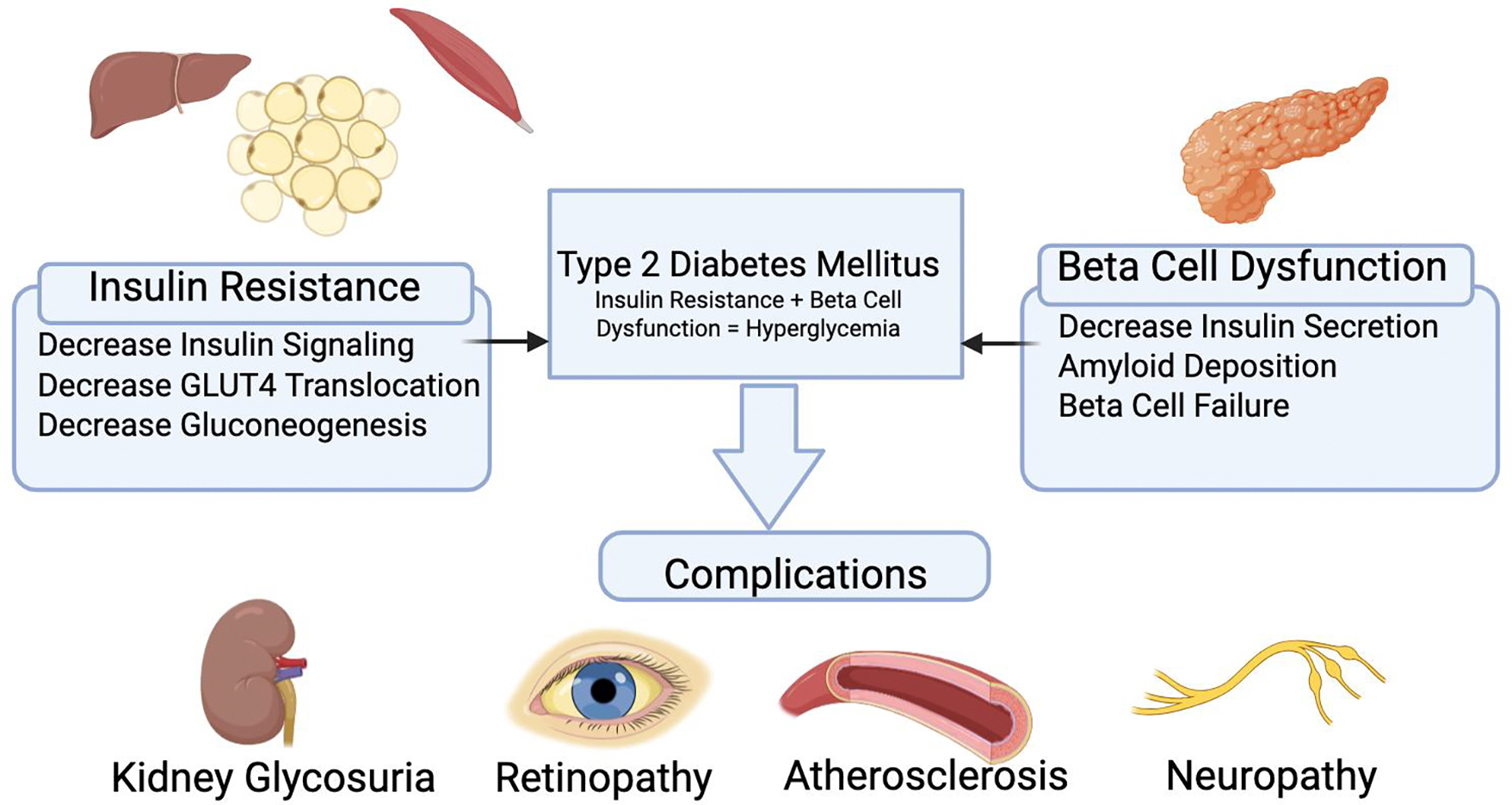
Pathogenesis of Type 2 Diabetes Mellitus and its Complications. This diagram illustrates the pathophysiology of Type 2 Diabetes Mellitus. It shows two main processes: insulin resistance in peripheral tissues (including adipose tissue, muscle, and liver) and β-cell dysfunction in the pancreas. On the left, adipose and muscle tissue demonstrate reduced insulin signaling, decreased GLUT4-mediated glucose uptake, and increased hepatic glucose production. On the right, the pancreas shows progressive β-cell dysfunction with decreased insulin secretion and amyloid deposition. Both pathways converge to cause hyperglycemia, which leads to downstream complications including kidney involvement (glycosuria), retinopathy, neuropathy, and atherosclerosis.

Type 2 Diabetes Mellitus is a chronic condition in which the body is unable to effectively regulate blood glucose levels due to insulin resistance and a progressive decline in insulin production. Insulin is a hormone responsible for facilitating the uptake of glucose from the bloodstream into cells for energy utilization. When this process becomes impaired, glucose accumulates in the blood, resulting in hyperglycemia [13]. Insulin resistance and beta-cell dysfunction are central to the pathophysiology of Type 2 Diabetes (Figure 1). Insulin resistance occurs when target tissues, particularly muscle, adipose tissue, and the liver, fail to respond adequately to insulin. As a result, glucose uptake is reduced and blood glucose levels rise [7,14].

In the early stages, the pancreas compensates by increasing insulin secretion to maintain normal glucose levels. However, chronic metabolic stress leads to progressive deterioration of pancreatic beta-cell function. Over time, the pancreas becomes unable to produce sufficient insulin to overcome insulin resistance [15]. This combination of persistent insulin resistance and declining insulin production drives the progression of Type 2 Diabetes, leading to worsening hyperglycemia and increased risk of long-term complications (Figure 1).

### Risk Factors for T2DM

Type 2 Diabetes Mellitus develops due to multiple contributing factors, with genetic predisposition playing a significant role in determining an individual’s risk. The occurrence of this condition varies widely across different populations around the world, often influenced by ethnicity and geographic location. For example, higher rates have been observed among Japanese, Hispanic, and Native American populations [13]. More than 100 genetic loci have been identified as being associated with Type 2 Diabetes Mellitus, along with additional loci that influence related traits such as insulin resistance and blood glucose levels. Despite these discoveries, no single genetic variant common across different populations has been shown to have a strong impact on disease risk. The most significant variant identified so far is the rs7903146 polymorphism in the TCF7L2 gene, although its effect is still relatively modest, increasing the risk of developing T2DM by about 1.4 times [16].

Obesity is also a risk factor for Type 2 Diabetes. When someone has too much body fat, it releases substances that make cells less sensitive to insulin. This is called insulin resistance. When cells ignore insulin, sugar stays in the blood instead of being used for energy, leading to high blood sugar. This affects the pancreas because, since the cells are not responding well to insulin, the pancreas must make extra insulin to keep blood sugar normal. Over time, the pancreas becomes overworked and cannot make enough insulin, causing Type 2 Diabetes to develop [1–6,17].

Obesity also causes inflammation. Fat tissue is not just storage for energy; it also releases inflammatory chemicals called cytokines. Chronic inflammation can damage blood vessels, kidneys, and the heart, which makes diabetes complications worse [18]. Since obesity often comes with high blood pressure and abnormal cholesterol levels, it increases the risk of kidney disease and heart problems, which are part of what cardiovascular-kidney-metabolic syndrome describes [3,7,19]. Controlling weight through diet, exercise, or medication can reduce the risk of Type 2 Diabetes and improve heart and kidney outcomes. Drugs like Finerenone work alongside these efforts to help protect organs that can be damaged by obesity and diabetes [20].

### Pathophysiology of Kidney Damage in T2DM

One of the most serious complications of Type 2 Diabetes Mellitus is diabetic kidney disease, also known as diabetic nephropathy. This condition develops gradually as prolonged hyperglycemia damages the small blood vessels in the kidneys, specifically the glomeruli, which are responsible for filtering waste from the blood. High glucose levels lead to increased pressure within these filtering units, causing structural damage over time [21]. This damage reduces the kidneys’ ability to properly filter waste products and leads to protein leakage into the urine, a condition known as albuminuria. This is often one of the earliest detectable signs of kidney damage in patients with T2DM [22].

Additionally, hyperglycemia activates several harmful biochemical pathways, including the formation of advanced glycation end products, activation of protein kinase C, and increased oxidative stress [9, 23–29]. These processes contribute to inflammation and fibrosis within kidney tissue, ultimately leading to chronic kidney disease. Over time, this progression can result in end-stage renal disease, which requires dialysis or kidney transplantation. These mechanisms highlight why treatments such as finerenone, which targets inflammation and fibrosis, are important in slowing disease progression [30].

### Cardiovascular Complications in T2DM

Cardiovascular disease is the leading cause of death among individuals with Type 2 Diabetes Mellitus. Chronic hyperglycemia contributes to the development of atherosclerosis, a condition in which plaque accumulates in the arteries, restricting blood flow [3,7]. This process occurs due to endothelial dysfunction, inflammation, and lipid abnormalities that are commonly present in patients with T2DM. As a result, individuals with diabetes have a significantly higher risk of heart attacks, strokes, and heart failure compared to those without the disease [31].

Another key factor linking T2DM to cardiovascular disease is insulin resistance. When cells become resistant to insulin, the body compensates by producing more insulin, which can contribute to hypertension and abnormal lipid metabolism. Elevated triglyceride levels and reduced levels of high-density lipoprotein (HDL) cholesterol further increase cardiovascular risk [7,32].

These interconnected risk factors contribute to the development of Cardiovascular-Kidney-Metabolic syndrome, a condition in which metabolic, kidney, and cardiovascular disorders overlap and exacerbate one another.

### Role of Oxidative Stress in Disease Progression

Another key factor that contributes to the complications of Type 2 Diabetes Mellitus is oxidative stress. Oxidative stress is a condition that arises from an imbalance between harmful molecules called reactive oxygen species (ROS) and the ability of the body to fight back against these harmful molecules with antioxidants [33].

This contributes to a wide range of complications such as kidney and cardiovascular complications. Oxidative stress contributes to complications in the cardiovascular system by damaging the lining of the blood vessels, leading to endothelial dysfunction and thus the inability of the vessels to relax properly. This contributes to high blood pressure and atherosclerosis [34]. Oxidative stress contributes to complications in the kidneys by damaging the filtering units of the kidneys and thus the progression of diabetic nephropathy. Since oxidative stress is involved in multiple pathways, treatments that reduce inflammation and tissue damage, like finerenone, can help slow down these harmful effects [35].

### Role of RAAS Dysregulation

One of the key systems involved in the progression of Type 2 Diabetes Mellitus is the RAAS (which regulates blood pressure, fluid balance, and vascular function). In patients with T2DM, this system often becomes overactivated, leading to harmful effects on both the kidneys and cardiovascular system. Increased RAAS activity raises levels of angiotensin II and aldosterone, which cause blood vessels to constrict, increase blood pressure, and promote sodium retention [36]. Over time, these effects place significant stress on the kidneys by increasing pressure within the glomeruli, accelerating damage and contributing to the progression of chronic kidney disease. At the same time, RAAS overactivation contributes to structural and functional changes in the heart, including hypertrophy and fibrosis, which increase the risk of heart failure and other cardiovascular complications [37].

In addition to its effects on blood pressure, RAAS plays a major role in promoting inflammation and fibrosis, which are central to CKM syndrome. Aldosterone stimulates the production of pro-inflammatory cytokines and profibrotic factors that lead to tissue scarring in both the kidneys and heart [38]. This scarring reduces the ability of these organs to function properly over time. In the kidneys, fibrosis decreases filtration capacity, while in the heart, it leads to stiffening of the cardiac muscle and impaired pumping ability. These processes are not isolated; instead, they interact with other metabolic and inflammatory pathways, creating a cycle of worsening damage that drives disease progression. This explains why simply controlling blood glucose levels is often not enough to prevent complications in patients with T2DM. Traditional treatments targeting RAAS, such as ACE inhibitors and angiotensin receptor blockers (ARBs), have been widely used to reduce blood pressure and slow kidney damage. GLP-1 agonists and other incretins have also been reported to provide protective effects in cardio-renal diseases [39,40]. Cardioprotection has also been achieved with ischemic reconditioning and stem cell therapy [41–43].

While these therapies are effective, they do not fully block the effects of aldosterone, a phenomenon known as “aldosterone escape,” where aldosterone levels rise again despite treatment [44]. This limitation leaves patients at continued risk for inflammation and fibrosis. Finerenone addresses this gap by directly blocking the mineralocorticoid receptor, which is activated by aldosterone. By doing so, it provides an additional layer of protection beyond traditional RAAS inhibitors, specifically targeting the pathways responsible for tissue damage.

### Finerenone

Finerenone (brand name - Kerendia) is a non-steroidal mineralocorticoid receptor antagonist that targets pathways involved in inflammation, fibrosis, and maladaptive remodeling in the heart and kidneys [24]. Finerenone reduces cardiovascular outcomes and slows kidney disease progression in patients with chronic kidney disease and type 2 diabetes. Elevated potassium (hyperkalemia) is a potential risk, especially in patients with CKD [45].

Finerenone targets these metabolic pathways that are disrupted by the chronic illness by blocking mineralocorticoid receptor overactivation reducing inflammation and fibrosis [46]. Finerenone is a non-steroidal mineralocorticoid receptor antagonist with high selectivity which is used for patients that have T2DM along with chronic kidney disease. Finerenone is marketed under the brand name Kerendia and is available in 10 mg and 20 mg tablet formulations.

**Figure 2:**
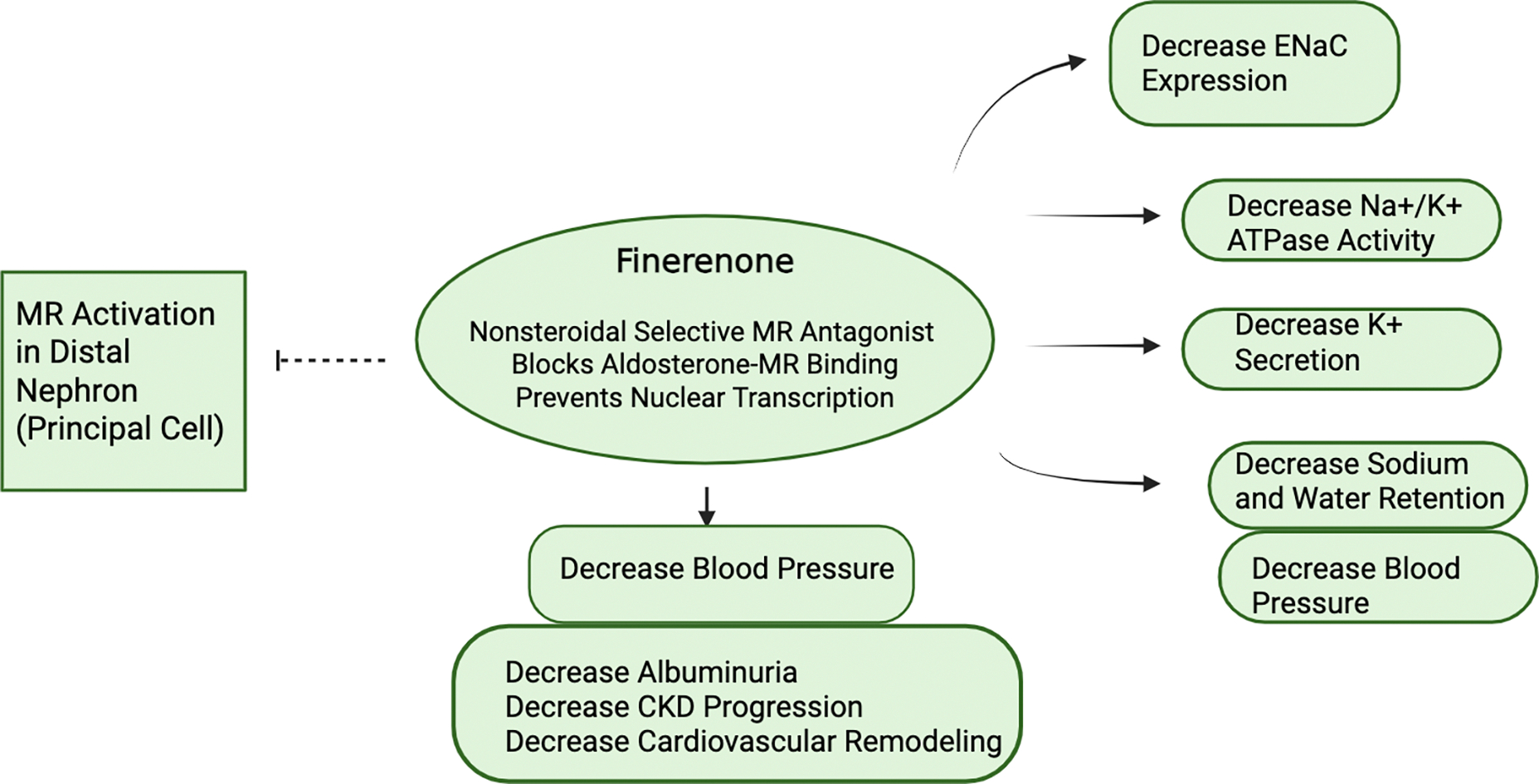
Mechanism of Action of Finerenone in the Distal Nephron. Finerenone, a nonsteroidal selective mineralocorticoid receptor antagonist, blocks aldosterone-mediated MR activation in principal cells of the distal nephron. This inhibits transcriptional upregulation of epithelial sodium channels (ENaC) and Na^+^/K^+^-ATPase, leading to reduced sodium reabsorption and decreased potassium excretion. Downstream effects include reduced sodium and water retention, lower blood pressure, and attenuation of kidney and cardiovascular damage, resulting in decreased albuminuria and slower progression of chronic kidney disease.

Under normal conditions, aldosterone binds to mineralocorticoid receptors in kidney cells, especially in the distal nephron (distal tubules and collecting ducts) and increases the expression of proteins such as the epithelial sodium channel (ENaC) and the Na^+^/K^+^-ATPase pump (Figure 2). This leads to increased sodium reabsorption back into the bloodstream and potassium excretion into the urine. By blocking the mineralocorticoid receptor, finerenone reduces the expression and activity of these sodium transport pathways. As a result, less sodium is reabsorbed and more is excreted in the urine (natriuresis), which helps lower blood volume and blood pressure. At the same time, potassium excretion is reduced, which can increase serum potassium levels [47].

Beyond its effects on sodium and potassium balance, finerenone also works at the cellular level in the heart and kidneys. Overactivation of mineralocorticoid receptors promotes inflammation and fibrosis by increasing the production of pro-inflammatory cytokines and profibrotic factors. Finerenone however works by blocking the mineralocorticoid receptor which reduces inflammation and scarring in the heart and kidneys. This helps slow the progression of kidney damage and lowers the risk of major heart problems [48]. Studies have also shown that finerenone lowers protein levels in the urine (albuminuria) and slows the loss of kidney function, making it an important treatment for patients with T2DM and chronic kidney disease [49] (Figure 2).

Compared with traditional steroidal MRAs, finerenone has a lower affinity for other steroid hormone receptors, which reduces the likelihood of off-target hormonal side effects such as gynecomastia which is a known result of spironolactone use. This distinguishes it from agents such as spironolactone and eplerenone. Despite this improved selectivity, finerenone retains its function as a potassium-sparing diuretic [50].

### Safety and Side Effects of Finerenone

Though finerenone offers several benefits, it is also crucial to understand the possible risks that come with it. Among the major risks associated with finerenone is hyperkalemia, a medical condition where the potassium level in the blood is too high. This is a major concern, especially because potassium plays a crucial role in the functioning of the heart, and abnormal levels can lead to abnormal heart rhythms [48]. However, studies have shown that the risks of hyperkalemia are lower when using finerenone compared to other mineralocorticoid receptor antagonists. Therefore, with proper management of the level of potassium and the functioning of the kidneys, it is possible to manage the risks of hyperkalemia when using finerenone [51].

Other side effects are generally mild, making finerenone a relatively safe option for long-term treatment. Understanding these risks is important so that healthcare providers can balance the benefits and ensure safe use of the medication.

### Evidence from Clinical Trials Supporting the Efficacy of Finerenone

The effectiveness of finerenone in managing kidney disease and related complications has been demonstrated in various clinical trials. These clinical trials include the FIDELIO-DKD and FIGARO-DKD studies. The FIDELIO-DKD trial was conducted on patients with advanced chronic kidney disease and Type 2 Diabetes Mellitus. The results showed that finerenone reduces the incidence of kidney failure, a decline in eGFR by ≥40%, and end-stage kidney disease. In addition, the drug reduces levels of albuminuria, which is an important factor in the progression of kidney disease. The FIGARO-DKD trial, on the other hand, was on patients with earlier stages of chronic kidney disease. The results showed that finerenone reduces cardiovascular events such as myocardial infarction, stroke, and hospitalization for heart failure. In addition, the drug reduces cardiovascular mortality and non-fatal cardiovascular events. The results show that finerenone not only reduces the progression of kidney disease but also reduces cardiovascular mortality and non-fatal cardiovascular events [52,53].

These trials provide strong evidence that finerenone is effective across different stages of disease progression. Importantly, the results highlight its dual benefit in protecting both kidney and cardiovascular health, which is especially valuable in patients with CKM syndrome [54]. While hyperkalemia was observed as a side effect, it was generally manageable with proper monitoring, reinforcing the overall safety profile of the drug [55, 56].

### Clinical Effects of Finerenones Compared to other Emerging Therapies

Many emerging therapies fall short in preventing long-term organ damage, whereas Finerenone provides more comprehensive protection by blocking mineralocorticoid receptor-mediated injury. IL-6 inhibition involves monoclonal antibodies that target the IL-6 ligand, blocking a central inflammatory pathway linked to atherosclerosis, kidney decline, and cardiovascular events [57]. Early-phase studies show that IL-6 inhibition can significantly reduce inflammatory markers in patients with chronic kidney disease (CKD) and high cardiovascular risk [58]. However, IL-6 inhibitors are associated with side effects such as increased infection risk, injection site irritation, and potential neutropenia [59].

CETP inhibitors lower low-density lipoprotein (LDL) cholesterol and apolipoprotein B while raising high-density lipoprotein (HDL) cholesterol in patients with hyperlipidemia [60]. Although clinical trials show significant reductions in LDL-C and ApoB, outcome data, such as reductions in heart attacks or improvements in kidney outcomes, remain limited or unpublished [61,62]. As a result, there is no definitive evidence yet that these therapies reduce mortality or major cardiovascular events.

ANGPTL3 inhibition targets lipid metabolism by reducing triglycerides and LDL-C independently of LDL receptor activity. While it shows promise as a lipid-lowering therapy, large-scale outcome trials evaluating its effects on kidney and cardiovascular outcomes are still limited [63].

Overall, these therapies represent promising emerging treatments for patients at risk of cardiovascular, kidney, or metabolic diseases. However, compared to them, Finerenone currently has the strongest clinical evidence supporting its ability to improve both heart and kidney outcomes. While the other therapies are innovative, their long-term benefits remain less established.

### Stages of CKM Syndrome

**Figure 3:**
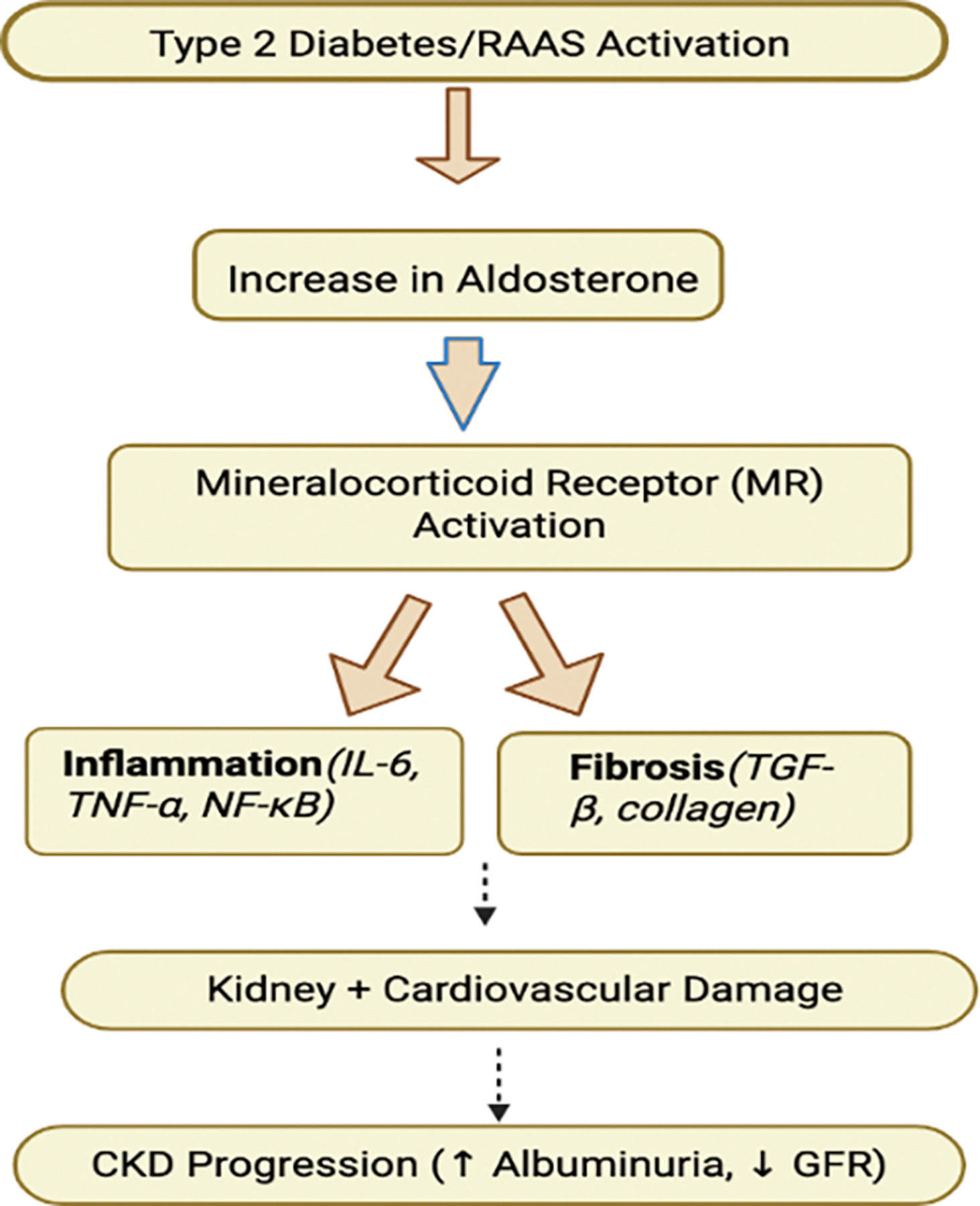
Pathophysiologic Role of Mineralocorticoid Receptor Activation in Cardiorenal Disease. Type 2 diabetes and RAAS activation increase aldosterone levels, resulting in activation of the mineralocorticoid receptor. MR activation promotes proinflammatory (IL-6, TNF-α, NF-κB) and profibrotic (TGF-β, collagen) pathways, which contribute to kidney and cardiovascular damage. These processes ultimately drive progression of chronic kidney disease, characterized by increased albuminuria and declining glomerular filtration rate.

CKM syndrome has different stages that start from risk factors and end with severe health complications. At the initial stages of the condition, the patient may present with risk factors such as obesity, high blood pressure, and insulin resistance without evidence of end-organ damage. As the condition advances to the next stages, the patient may start to show signs of metabolic disorders, kidney damage, and cardiovascular complications [64] (Figure 3).

In the later stages, the patient may also show signs of severe chronic kidney disease, heart failure, and severe cardiovascular complications such as heart attacks and strokes. The stages of the condition are interconnected, in the sense that the progression of one stage may accelerate the progression of the others. For instance, kidney damage may lead to high blood pressure, which may result in heart disease complications. Understanding these stages is important because early intervention can slow or prevent progression to more severe outcomes [65].

### Involvement of Multi-system Pathways in CKM Syndrome

CKM syndrome involves interacting biological pathways that contribute to disease development and progression. These pathways include inflammatory pathways, metabolic pathways, the RAAS system, oxidative stress pathways, and fibrosis pathways (Figure 3). Together, they create a multi-system network that drives progression and worsens outcomes in CKM syndrome [66].

**Figure 4:**
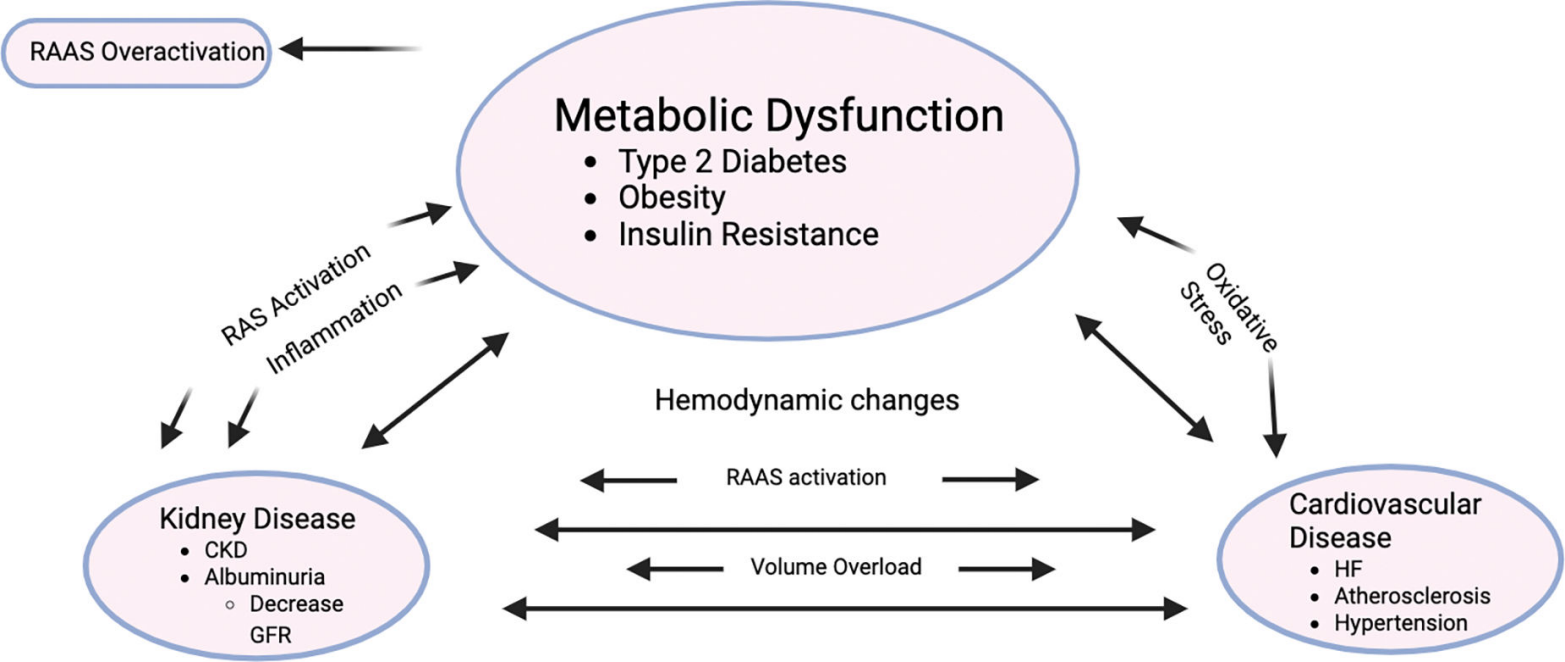
Cardio-Kidney-Metabolic (CKM) Syndrome. Metabolic dysfunction, chronic kidney disease, and cardiovascular disease are interconnected conditions that contribute to one another through shared mechanisms, including inflammation, RAAS activation, oxidative stress, and hemodynamic changes. These interactions promote disease progression across organ systems. Targeted therapies, such as finerenone, help interrupt these pathways and improve clinical outcomes, including reduced CKD progression, heart failure hospitalization, and cardiovascular mortality.

One of the most important aspects to understand in Type 2 Diabetes Mellitus (T2DM) and CKM syndrome is the interconnectedness of the body’s systems (Figure 4). The metabolic, kidney, and cardiovascular systems are not separate; they constantly influence one another, and problems in one system inevitably cause problems in another. In T2DM, elevated blood glucose levels trigger a chain reaction affecting several organs simultaneously [67].

For instance, prolonged high blood glucose negatively affects blood vessels, which strains the kidneys and increases the risk of atherosclerosis and hypertension in the heart. At the same time, kidney strain can lead to hypertension, which further affects the heart. This creates a cycle where all three conditions worsen each other [68].

Hormones and pathways also play a critical role in this interconnection. The RAAS system, inflammatory pathways, and metabolic pathways interact with one another. If one pathway becomes overly active, it can trigger another, causing widespread damage. For example, insulin resistance increases blood sugars and leads to increased inflammation and oxidative stress, contributing to both kidney and cardiovascular disease [69].

Due to this close association, it is not enough to simply reduce blood sugar levels, as heart and kidney damage may still occur due to inflammation and fibrosis. Newer drugs like finerenone address multiple mechanisms simultaneously; for example, finerenone reduces inflammation and fibrosis, breaking the vicious cycle between kidney and cardiovascular disease [70].

Hypertension, or high blood pressure, is another key factor. It is very common in patients with T2DM, affecting blood vessels, the heart, and the kidneys. It increases glomerular pressure in the kidneys, hastening kidney function decline, and thickens heart muscles, which may eventually cause heart failure [71].

Lipids, or blood fats, are also important. Many T2DM patients have abnormal cholesterol levels, including increased LDL and decreased HDL. These lipid imbalances can form arterial plaques, leading to heart attacks and strokes, and can exacerbate kidney damage through inflammation and oxidative stress [72].

Inflammation is another critical link between systems. Low-grade inflammation is common in patients with obesity and T2DM, affecting nearly all organs. It contributes to blood vessel, kidney, and heart damage, worsening complications even when blood sugar levels are somewhat controlled [73].

The final stage of damage across these pathways is fibrosis, or scarring. The body responds to repeated injury from high blood sugars, inflammation, and oxidative stress by scarring affected areas. Excessive scarring replaces functional tissue with non-functioning tissue, impairing kidney filtration and the heart’s pumping ability [74].

Therefore, fibrosis is such an integral target in treatment. Finerenone specifically reduces scarring, protecting both the kidneys and the heart. By slowing fibrosis, the drug extends organ function and reduces the risk of serious complications [75].

Overall, the combination of the metabolic, kidney, and cardiovascular systems explains the complexity and difficulty of treating CKM syndrome. The interaction between these systems creates a cycle of damage that is hard to break once established, emphasizing the importance of early intervention and multi-targeted therapy in patients with Type 2 Diabetes Mellitus.

### Importance of Personalized Medicine

With the advancement of research in the field of medicine, the importance of personalized medicine is increasing in the treatment of complex diseases such as Type 2 Diabetes Mellitus. Personalized medicine is a medical approach that focuses on the genetic characteristics and lifestyle of an individual and the risk factors that may affect the patienťs health. This approach enables the doctor to select the best medicine for the patient rather than a generalized approach [76].

For instance, there are patients who might benefit more from medications that act on blood sugar, while others might benefit from medications that act on the kidneys or heart. Finerenone is particularly useful in patients who are at high risk of CKD or cardiovascular disease. By integrating both personalized medicine and targeted therapies, healthcare providers can improve outcomes and reduce complications in patients with T2DM [77].

## Discussion

The results suggest that finerenone is effective because it targets the underlying mechanisms of disease rather than only the symptoms. Finerenone blocks the processes that high blood sugar leads to which explains why it protects both the kidneys and the heart. By blocking these processes, it explains why it can protect both the kidneys and heart at the same time [78]. This effect is especially important in CKM syndrome where multiple organ systems are affected together [79].

### Economic and Public Health Impact

Type 2 Diabetes Mellitus also affects the economy and the healthcare system. The cost of managing diabetes and the complications associated with it is extremely high. In the USA alone, billions of dollars are spent annually on managing diabetes and diabetes-related complications. This includes the cost of medication, hospitalizations, and long-term care. The cost includes managing the disease and the complications that occur. The complications may include kidney failure and cardiovascular disease. These may require treatments such as dialysis and surgeries. These medications, by slowing down the disease process and thereby preventing complications, may help reduce the cost of managing the disease. This not only increases the quality of life for the patients but also reduces the burden on the healthcare system [80].

### Lifestyle Interventions

While pharmacological treatments like finerenone are critical for protecting the kidneys and heart in patients with Type 2 Diabetes Mellitus, lifestyle interventions remain a cornerstone of long-term management. Diet, physical activity, and weight control play essential roles in reducing insulin resistance, controlling blood sugar, and improving overall cardiovascular and kidney health [81]. A diet rich in whole grains, vegetables, fruits, and lean protein, while low in processed sugars and saturated fats, helps manage blood glucose and cholesterol levels, lowering the risk of complications.

Regular exercise, including both aerobic and resistance training, enhances insulin sensitivity and helps reduce body fat, which in turn decreases inflammation and lowers blood pressure [82]. Weight management not only improves metabolic control but also reduces the workload on the heart and kidneys, complementing the effects of medications like finerenone.

In addition, patient education and behavioral support are important for maintaining adherence to both lifestyle changes and medication regimens. Programs that teach patients about nutrition, exercise, and self-monitoring of blood glucose have been shown to improve outcomes and reduce the risk of complications [83]. Together, lifestyle interventions and targeted pharmacotherapy offer a comprehensive approach to managing T2DM and preventing the progression of CKM syndrome.

### Limitations

Despite these strong results, most studies focus on adults with advanced diseases rather than early-stage patients. Long term effects are still not fully uncovered. Along with this, there is limited data on diverse populations such as for younger patients [84].

Although these medications are effective in treating Type 2 Diabetes Mellitus, one of the major challenges in the management of Type 2 Diabetes Mellitus is patient adherence to treatment regimens. Adherence is defined as the extent to which patients follow their medication regimens, make lifestyle changes, and observe advice from healthcare providers. Patients often fail to take their medication regularly, eat a balanced diet, and exercise regularly. This may impair the efficacy of these regimens and ultimately contribute to poor health outcomes [85,86].

One reason for this difficulty in compliance is that T2DM is a long-term condition that needs to be managed continuously. The patient might get frustrated by having to take several drugs or make significant lifestyle changes. Sometimes, the side effects or costs of the medication might also act as a deterrent for the patient [87].

Adherence is a key factor that needs to be addressed to obtain the best possible benefits from drugs such as finerenone. This can be achieved by providing the patient with proper education and care. When a patient understands the importance of the treatment and the condition they are in, they can be more consistent with the treatment regimen, thus improving the health of the kidneys and the heart [88].

### Predictions

Future research will likely focus on long term effectiveness of finerenone and use in earlier stages of disease. Research will most likely also begin expanding treatment to broader patient populations to gain more results [89].

## Conclusion

Type 2 Diabetes Mellitus is a complex and increasingly common disease that affects multiple organ systems, especially the kidneys and cardiovascular system. Its progression is driven by interconnected factors such as insulin resistance, chronic inflammation, and metabolic dysfunction, which contribute to the development of CKM syndrome. These overlapping conditions significantly increase the risk of severe complications, including chronic kidney disease and cardiovascular events. As a result, treatments that address the underlying causes of damage rather than just symptoms are essential for improving patient outcomes.

Finerenone has emerged as an important therapy because it directly targets key pathways involved in inflammation and fibrosis. By blocking the mineralocorticoid receptor, it helps protect both the kidneys and heart, slowing disease progression and reducing the risk of major complications

Although additional research is needed to better understand its long-term effects and effects in broader populations, current evidence supports finerenone as a key advancement in the treatment of CKM syndrome. Moving forward, combining therapies like finerenone with other emerging treatments and early intervention strategies may further improve outcomes. Overall, finerenone represents a significant step toward an effective and targeted management of Type 2 Diabetes and its complications by helping reduce the growing global burden of this disease.

## Key Points

- Type 2 Diabetes Mellitus (T2DM) is a progressive multisystem disease driven by insulin resistance and β-cell dysfunction, leading to chronic hyperglycemia.
- The prevalence of T2DM is rising globally and represents a major public health burden due to its long-term health complications.
- Chronic hyperglycemia contributes to endothelial dysfunction, oxidative stress, inflammation, and vascular injury.
- T2DM significantly increases the risk of chronic kidney disease and cardiovascular disease through overlapping pathophysiologic mechanisms.
- Activation of the renin–angiotensin–aldosterone system (RAAS) plays a central role in promoting hypertension, fibrosis, and organ damage in CKM syndrome.
- Diabetic kidney disease develops from progressive glomerular injury, leading to albuminuria and eventual decline in renal function.
- Cardiovascular complications in T2DM are largely driven by atherosclerosis, lipid abnormalities, and persistent insulin resistance.
- Finerenone, a non-steroidal mineralocorticoid receptor antagonist, reduces inflammation and fibrosis, providing cardio-renal protection in T2DM with CKD.
- Clinical trials (FIDELIO-DKD and FIGARO-DKD) demonstrate that finerenone slows kidney disease progression and reduces cardiovascular events.
- Effective management of T2DM requires targeting metabolic, inflammatory, and hormonal pathways to improve long-term outcomes in CKM syndrome.
